# Psychological impact of armed conflict and forced displacement on children and adolescents

**DOI:** 10.3389/frcha.2026.1912159

**Published:** 2026-09-02

**Authors:** Maryam Masoomi, Mahdieh Saeidi, Mona Salehi, Sakshi Prasad, Omar Alzein, Deblina Roy, Mohita Joshi, Sasidhar Gunturu

**Affiliations:** 1 Department of Psychiatry, Tehran University of Medical Sciences, Tehran, Iran; 2 Department of Psychiatry, BronxCare Health System, Bronx, NY, United States; 3 Department of Psychiatry, University of Minnesota, Minneapolis, MN, United States; 4 Department of Psychiatry, King George’s Medical University, Lucknow, Uttar Pradesh, India; 5 Department of Psychiatry, Era’s Medical College, Lucknow, Uttar Pradesh, India

**Keywords:** armed conflict, child and adolescent mental health, forced displacement, psychological trauma, PTSD, trauma-informed care

## Abstract

**Introduction:**

Armed conflict and forced displacement disrupt the safety, caregiving, education, and healthcare essential for healthy child development, placing children and adolescents at increased risk for adverse mental health outcomes. This narrative review summarizes the psychological consequences of war and displacement and highlights trauma-informed interventions across different stages of recovery.

**Methods:**

A narrative review of the literature was conducted using PubMed and Google Scholar. Studies published between January 2000, and January 2025 were identified using keywords related to children, adolescents, armed conflict, war, forced displacement, refugee populations, mental health outcomes, post-traumatic stress disorder (PTSD), depression, anxiety, and psychosocial interventions. Relevant English-language articles addressing psychological outcomes and intervention strategies among conflict-affected youth were reviewed and synthesized.

**Results:**

Across studies, exposure to armed conflict and displacement was consistently associated with elevated rates of PTSD, depression, anxiety, behavioral disturbances, sleep problems, and impaired social functioning. Mental health effects were observed during acute crises and often persisted long after conflict exposure, increasing the risk of chronic psychiatric disorders and intergenerational transmission of trauma. Protective factors included stable caregiving relationships, educational continuity, social support, and access to community-based psychosocial services. However, significant disparities in service availability and accessibility were reported, particularly among displaced and refugee populations.

**Conclusion:**

Armed conflict and forced displacement harm children through direct trauma exposure, disrupted caregiving, loss of educational and social structure, chronic deprivation, and barriers to care, with effects that persist well beyond the acute phase. Trauma-focused cognitive behavioral therapy and narrative exposure therapy have the strongest supporting evidence but are the least scalable in active conflict, whereas group and non-specialist approaches offer reach with more variable effects. Intergenerational transmission is best supported for psychological, familial, and social pathways; biological mechanisms remain preliminary. Stepped, layered, and culturally adapted delivery embedded in schools, families, and primary care is needed.

## 1 Introduction

Healthy psychosocial development in childhood relies on the fulfillment of fundamental human rights, including physical safety, emotional security, stable caregiving relationships, and access to healthcare and education. Under typical conditions, childhood represents a critical period of cognitive, emotional, and social growth, supported by protective family and community structures. Armed conflict, however, systematically dismantles these foundations for millions of children worldwide, exposing them to profound adversity and placing them at heightened risk for both immediate and enduring psychological harm (1).

Children and adolescents affected by war and forced displacement are disproportionately exposed to violence and instability, including direct injury, family separation, forced recruitment, sexual violence, and attacks on schools and healthcare facilities. These experiences occur during sensitive developmental windows, compounding vulnerability and disrupting normative emotional, cognitive, and social trajectories (2). Beyond direct exposure to violence, the destruction of infrastructure, interruption of education, erosion of social support systems, and loss of caregivers further magnify psychological distress and undermine resilience (3–5).

The global scale of this crisis has reached unprecedented levels. According to UNICEF analyses of recent global trends, nearly 19% of the world's children, more than 473 million were living in conflict-affected settings in 2024, a dramatic increase from approximately 10% in the 1990s and the highest proportion recorded since World War II (6). The same year is projected to set a record for United Nations–verified grave violations against children, with nearly 33,000 violations affecting over 22,000 children documented in 2023 alone (6). Concurrently, forced displacement continues to escalate, with more than 47 million children displaced by violence as of 2023, a figure expected to rise further as conflicts intensify (7). For many children, displacement entails prolonged instability, separation from caregivers, and exposure to unsafe living conditions, all of which carry lasting psychological consequences. Recent conflicts continue to demonstrate the devastating impact of war on children and families. Reports from the 2026 Iran conflict described attacks affecting schools, including a reported strike on a girls’ school in Minab that resulted in approximately 180 child fatalities. Beyond the tragic loss of young lives, such events can leave families and caregivers with prolonged grief, trauma, and psychological distress (8).

Armed conflict also creates severe humanitarian conditions characterized by food insecurity, limited access to clean water, inadequate shelter, and disruption of essential healthcare services. These stressors intersect with entrenched poverty, as conflict-affected countries experience substantially higher rates of economic deprivation compared to non-conflict settings (9). Such conditions not only increase mortality and physical morbidity but also exacerbate emotional distress, heighten the risk of mental health disorders, and constrain opportunities for recovery and development.

A growing body of evidence demonstrates that children exposed to armed conflict and forced displacement face elevated rates of post-traumatic stress disorder (PTSD), depression, anxiety, behavioral dysregulation, and impaired social functioning (4, 10–12). While some effects emerge immediately, others persist across adolescence and into adulthood, influencing educational attainment, economic stability, interpersonal relationships, and overall quality of life. Moreover, emerging research suggests that the psychological impact of war may extend beyond directly exposed individuals, contributing to intergenerational trauma through altered stress regulation, family dynamics, and sociocultural transmission of adversity (13).

Despite increasing recognition of the substantial mental health burden borne by conflict-affected children and adolescents, access to effective, developmentally appropriate, and culturally responsive care remains limited and unevenly implemented.

This narrative review examines the psychological impact of armed conflict and forced displacement on children and adolescents across immediate, short-term, and long-term timeframes. It synthesizes evidence on mental health outcomes, explores the mechanisms through which conflict disrupts development, and highlights the role of caregiving, education, and community structures in shaping resilience and recovery. In addition, the review considers ethical, cultural, and structural challenges in trauma-informed care and identifies critical gaps in research and service delivery relevant to real-world humanitarian contexts.

### 1.1 Materials and methods

#### 1.1.1 Study design

This study was conducted as a narrative literature review examining the mental health impact of armed conflict and forced displacement on children and adolescents, with emphasis on psychological outcomes, developmental consequences, and trauma-informed interventions across conflict settings.

### 1.2 Search strategy

A focused literature search was conducted using PubMed and Google Scholar to identify relevant literature published between January 2000 and January 2025. PubMed was selected because it indexes high-quality, peer-reviewed biomedical and mental health research, while Google Scholar was used to broaden retrieval by capturing interdisciplinary publications and relevant articles not always indexed in PubMed. Search terms combined keywords related to (i) the population (child, children, adolescent, youth), (ii) exposures (war, armed conflict, political violence, conflict zones, forced displacement, refugees, internally displaced persons [IDPs]), and (iii) outcomes and interventions (mental health, psychological trauma, PTSD, depression, anxiety, psychosocial support, and trauma-informed care), using Boolean operators (AND/OR). Reference lists of relevant publications were also manually screened to identify additional studies.

### 1.3 Study selection and eligibility criteria

Studies were screened based on their titles, abstracts, and full texts for relevance to the psychological impact of armed conflict or forced displacement among children and adolescents and to trauma-informed interventions or psychosocial support. Quantitative, qualitative, mixed-methods studies, systematic reviews, meta-analyses, and narrative reviews published in English were considered eligible. Studies focusing exclusively on adults or not addressing mental health outcomes in children and adolescents were excluded. Because this was a narrative review, priority was given to high-quality, influential, and thematically relevant publications rather than exhaustive study identification. In addition to peer-reviewed literature, selected grey literature from internationally recognized organizations, including UNICEF, the World Health Organization (WHO), the United Nations (UN), and the International Committee of the Red Cross (ICRC), was included when it provided relevant epidemiological data, humanitarian guidance, or policy documents.

### 1.4 Data synthesis

Findings were synthesized narratively using a thematic approach, integrating evidence on immediate, short-term, and long-term psychological outcomes, intergenerational trauma, resilience factors, intervention strategies, and ethical considerations in trauma-informed care.

## 2 Findings

The findings are organized along two intersecting axes. The first is temporal, following the immediate, short-term, and long-term consequences of exposure. The second is mechanistic, and specifies five pathways through which armed conflict and forced displacement produce psychological harm: (i) direct trauma exposure, including injury, witnessed violence, bereavement, and forced participation in hostilities; (ii) disruption of caregiving, encompassing separation, caregiver death, caregiver mental illness, and the erosion of parenting capacity under survival conditions; (iii) loss of educational and social structure, including the destruction of schools, interrupted schooling, and the fragmentation of peer and community networks; (iv) chronic deprivation, spanning food insecurity, unsafe water, inadequate shelter, and entrenched poverty; and (v) barriers to care, arising from health system collapse, workforce scarcity, discontinuity of care during displacement, and stigma (5, 10, 17, 18). These mechanisms are not phase-specific. Each operates across the whole temporal arc and changes in character rather than resolving, so the sections below describe how displacement, caregiver separation, educational disruption, food insecurity, and healthcare collapse manifest differently at different points after exposure rather than treating them as discrete events belonging to discrete phases. Cross-cutting themes, including intergenerational transmission, education and poverty, resilience and equity, ethical considerations, and the role of stakeholders, follow the temporal sections. A summary across phases is presented in Table 1.

**Table 1:** Psychological impact of armed conflict and forced displacement on children and adolescents across temporal phases.

Timeframe	Primary stressors and exposures	Common psychological and developmental outcomes	Potential long-term consequences
Immediate Impact (during conflict and acute displacement)	• Direct exposure to violence (bombings, shelling, injury) • Forced displacement and loss of home • Family separation or caregiver loss • Food insecurity, lack of shelter, unsafe living conditions	• Acute stress reactions • Fear, hyperarousal, emotional distress • Sleep disturbances and somatic complaints • Regression, clinginess, aggression, or withdrawal	• Increased risk of PTSD onset • Disrupted attachment formation • Early emotional dysregulation
Short-Term Impact (months to a few years post-exposure)	• Prolonged displacement (refugee or IDP settings) • Disrupted education and healthcare • Ongoing insecurity and uncertainty • Caregiver psychological distress	• Post-traumatic stress disorder (PTSD) • Depression and anxiety disorders • Behavioral problems and impaired social functioning • Academic difficulties and developmental delays	• Persistence of mental health disorders • Impaired educational attainment • Increased vulnerability to substance use and risk-taking behaviors in adolescence
Long-Term Impact (adolescence into adulthood)	• Chronic stress exposure during development • Prolonged poverty and social marginalization • Unresolved trauma and loss • Limited access to long-term mental health care	• Chronic PTSD, depression, and anxiety • Impaired cognitive functioning and emotional regulation • Difficulties with relationships and social integration	• Reduced economic and educational opportunities • Intergenerational transmission of trauma • Increased risk of chronic illness and societal disengagement

PTSD, post-traumatic stress disorder; IDP, internally displaced person.

### 2.1 Immediate impact: direct trauma exposure and acute deprivation

The immediate impact of armed conflict on children and adolescents involves sudden and chaotic disruption of their lives, resulting in severe physical, psychological, and social challenges. Exposure to active conflict zones and forced displacement significantly heightens the risk to their overall well-being. Direct consequences include penetrating injuries, blunt trauma, crush injuries, and burns often caused by shelling, explosions, and other acts of violence (14). Armed conflict has been linked to a three-fold increase in infant and child mortality, largely due to the widespread lack of essential resources. Children in war-affected areas frequently face shortages of food, clean water, and adequate nutrition, which worsens malnutrition and weakens their immune systems, leading to elevated mortality rates and a rise in diseases, including acute malnutrition (15, 16). Additionally, overcrowded and unsanitary living conditions further increase the spread of infectious diseases, compounding their already critical health risks.

#### 2.1.1 Disruption of services and early barriers to care

The devastation of war extends far beyond the battlefield, dismantling critical infrastructure and essential services. Armed conflict leads to the collapse of healthcare systems, education, and social support structures, worsening outcomes for children and adolescents. Communities fracture, economies weaken, and access to necessities such as schooling and healthcare is severely disrupted (17). The shortage of medical supplies, trained personnel, and functioning healthcare facilities contributes to rising mortality and morbidity rates among children (15). Forced displacement further compounds these issues, particularly for children who are separated from their caregivers and adult protection. Many become “unaccompanied minors,” left to navigate life independently or in unstable environments such as refugee camps, orphanages, or with distant relatives. These conditions create significant psychological stress and increase vulnerability to mental health disorders (18).

#### 2.1.2 Psychosocial effects and disruption of caregiving

Children's responses to the trauma of war are closely shaped by their developmental stage. In many cases, caregivers are preoccupied with basic survival, leaving children to process distressing experiences without emotional support. This lack of guidance can disrupt attachment patterns and result in a range of psychological and behavioral responses, including heightened fear, aggression, dependency, and psychosomatic symptoms. Without timely intervention, these reactions can persist and hinder healthy emotional development. Targeted, developmentally appropriate psychosocial interventions are therefore essential to support children exposed to the trauma of war and forced displacement (19).

#### 2.1.3 Child soldiers

The recruitment of child soldiers remains one of the most alarming consequences of armed conflict. In many war-torn regions, children are forcibly or coercively drawn into military roles, placing them at severe risk of both inflicting and enduring harm (20). This experience adds another layer of trauma, compounding the already significant physical and psychological burdens faced by children in conflict zones (21). Understanding the immediate and long-term effects of such involvement is essential for developing effective support systems. A holistic, trauma-informed approach that addresses intersecting challenges, such as health, displacement, and disrupted services, is crucial to fostering resilience and supporting the recovery of these vulnerable children.

### 2.2 Short-term impact: protracted displacement and chronic deprivation

In the months and years following acute exposure, the same mechanisms persist but change form rather than resolve. Displacement becomes protracted rather than sudden, caregiver separation hardens into prolonged absence or permanent loss, and deprivation becomes chronic rather than episodic (17, 18). What distinguishes this phase is the added burden of adaptation, as children negotiate unfamiliar languages, cultures, legal statuses, and schooling systems at precisely the point when the family and community structures that would ordinarily support that adaptation are themselves degraded. Understanding this phase is essential for designing timely interventions that address urgent needs and mitigate long-term consequences.

#### 2.2.1 Health challenges in the short term and persisting barriers to care

The deprivations described in the preceding section become entrenched in this phase rather than intensifying acutely, and it is their persistence rather than their severity that distinguishes them. Displacement interrupts immunization schedules and continuity of primary care, contributing to excess child mortality well after active hostilities subside (15, 27). Maternal health also suffers, with refugee mothers facing higher risks of preterm birth, stillbirth, and adverse pregnancy outcomes (19, 27), which in turn degrades the caregiving environment available to surviving children and links deprivation back to the caregiving pathway.

#### 2.2.2 Education and psychosocial burden: loss of educational and social structure

Education is often interrupted or inaccessible because schools are destroyed, occupied, or unreachable following displacement (5, 14); the downstream consequences for poverty and social mobility are treated separately below to avoid duplicating them here. The psychosocial impact of losing this structure is equally profound. Fragmented family structures, parental separation, and exposure to violence increase the risk of mental disorders such as PTSD and depression conditions, especially prevalent among conflict-affected youth (22). Children, with fewer coping strategies than adults, are particularly susceptible to trauma. In the aftermath, adolescents may turn to substance use, while younger children often exhibit anxiety, fear, and behavioral changes. In severe cases, some are indoctrinated into violence, eroding their moral development. Caregivers’ mental health, often neglected, also plays a critical role in shaping children's recovery. The cumulative burden of displacement, poor health, and disrupted education underscores the urgent need for comprehensive, trauma-informed support systems to aid young people's healing and future development.

### 2.3 Long-term impact: cumulative effects across mechanisms

The long-term impact of war on children is profound, affecting their health, education, and societal roles in adulthood. These persistent challenges extend beyond the immediate aftermath of conflict and require interventions that address the enduring consequences of childhood war experiences.

#### 2.3.1 Enduring displacement challenges

The effects of armed conflict often persist into adulthood, manifesting as long-term challenges in resettlement, social integration, and overall well-being. Many children exposed to war continue to struggle with chronic mental health conditions such as PTSD, anxiety, and depression, alongside physical health issues and impaired social functioning (17, 22). Prolonged exposure to stress during early development can disrupt neural connections, adversely affecting learning, memory, and reasoning abilities. Disrupted education and weakened economic systems contribute to a cycle of poverty, further compromising health, nutrition, and future opportunities. In some contexts, constructs such as post-traumatic slavery syndrome have been proposed to describe an additional layer of intergenerational burden affecting identity, relationships, academic performance, and life satisfaction, although these remain descriptive formulations without validated measurement or prospective support (29, 32). The impact of war extends beyond clinical diagnoses, influencing multiple aspects of a child's developmental trajectory and quality of life.

#### 2.3.2 Perpetuation of violence and societal strain

The long-term consequences of war often perpetuate cycles of violence, particularly through the traumatic experiences of child soldiers. Post-conflict data reveal disturbing realities: nearly a quarter of former child soldiers report having killed someone, including family members, and a third of girls report having been raped (25). The “audience effect,” in which adolescents internalize violence as performance, further fuels ongoing aggression and gun violence in affected communities, embedding war's legacy into the next generation (26). The societal strain extends far beyond immediate mental health outcomes. Biological mechanisms have also been proposed. Differences in stress-related gene expression, and in DNA methylation at glucocorticoid-related loci, have been reported in trauma-exposed individuals and, in a smaller number of studies, in their offspring (23, 24). Whether such differences are transmitted through the germline, rather than mediated by prenatal exposure, postnatal caregiving, and shared social environment, remains unresolved (23, 61). These findings are therefore best treated as a candidate mechanism rather than a demonstrated inherited effect (23, 61). Post-conflict societies also face major health and environmental challenges. Millions of children with war-related disabilities lack access to rehabilitation services, while damaged infrastructure, contaminated environments, and scarce clean water continue to endanger public health (15, 27). Destroyed healthcare systems and disrupted prenatal care further complicate recovery, particularly for pregnant women and newborns.

### 2.4 Generational trauma

Research on generational trauma spans mechanisms that differ substantially in how well they are supported, and conflating them overstates what is currently known. The best-supported pathways are psychological, familial, and sociocultural. Parental post-traumatic symptoms, altered parenting behaviors, disrupted family communication, and the transmission of narratives and silences about the traumatic past are consistently associated with offspring adjustment across multiple populations and study designs (28, 29, 31, 65). Danieli's model describes how initial trauma, post-traumatic family adaptational styles, and the surrounding sociocultural environment interact across generations to shape both individual and collective recovery, and the associated inventory has been empirically developed and validated in offspring of survivors (28, 29). Reported patterns include a victim or fighter adaptational style, emotional numbness, and overprotectiveness in the exposed generation, with social withdrawal, difficulty trusting others, heightened anxiety, and difficulty perceiving others as safe described among their children (30). A systematic review reports that children exposed to war often experience sleep disturbance, adjustment difficulties, psychological distress, and later parenting challenges, while noting substantial heterogeneity across studies (31). Even within this better-supported tier, transmission is conditional rather than automatic and effect sizes are moderate: a meta-analysis of 32 samples of Holocaust survivor offspring found evidence of secondary traumatization in clinical samples but not in adequately designed non-clinical samples, indicating that offspring risk depends on the presence of additional stressors rather than following inevitably from parental exposure (62).

Biological pathways occupy a different evidential tier and should not be described in the same register. Prenatal and early postnatal programming, in which maternal stress during pregnancy and disrupted early caregiving influence offspring hypothalamic-pituitary-adrenal axis regulation, draws on convergent animal and human data and is best characterized as a plausible and partially established mechanism (23). Germline epigenetic inheritance in humans, by contrast, in which trauma-induced marks are transmitted through gametes independently of the postnatal environment, remains preliminary and actively contested. The most compelling demonstrations come from controlled animal models; human studies are small, largely cross-sectional, restricted to a narrow set of candidate loci, and unable to separate germline transmission from shared postnatal environment, cultural transmission, and genetic inheritance (23, 61). Claims of inherited epigenetic effects in war-exposed families should accordingly be read as hypotheses under investigation rather than as established findings, and neither clinical practice nor policy argument should rest on them. This review distinguishes throughout between established evidence, plausible mechanisms, and speculative hypotheses. The practical implication is unchanged by the uncertainty, since the modifiable targets identified by the well-supported psychological and social pathways, namely caregiver mental health, family functioning, and community support, are the same targets that intervention research has shown to be tractable (10, 64).

### 2.5 Education, social structures, and poverty

The long-term effects of trauma and crisis profoundly impact education, social cohesion, and poverty levels, often perpetuated across generations through intergenerational trauma (32). While trauma can be inherited, communities and individuals possess the capacity for healing and recovery (33). Education is a critical pathway to upward mobility (34), yet trauma can disrupt concentration, emotional regulation, and the ability to form trusting relationships, impairing learning outcomes (35, 36). Additional barriers such as financial instability, unstable housing, and lack of resources further limit access to education for trauma-affected populations (37). The economic consequences of conflict compound these barriers and are deeply entrenched, since sanctions, displacement, and infrastructure collapse generate chronic poverty that constrains access to education, healthcare, and employment across generations and makes recovery exceptionally difficult (34, 37). Children who grow up in these conditions face diminished opportunities, which calls for sustained interventions that address immediate needs while also interrupting the transmission of trauma, poverty, and vulnerability to the next generation (42).

Schools are increasingly adopting trauma-informed approaches that foster safe, supportive environments and equip educators with tools to recognize and respond to trauma (38, 39). These strategies create opportunities for healing and academic success. Likewise, while trauma can weaken social structures, communities have the potential to rebuild through investment in support services, social cohesion, and collective resilience (40, 41).

### 2.6 Resilience, agency, and equity

Despite trauma's far-reaching impact, resilience and personal agency are key to breaking cycles of poverty and psychological distress. Access to mental health care, social support programs, and inclusive policies empowers individuals and families to heal and thrive (42). Structural efforts to reduce inequality through affordable healthcare, education, and economic opportunities are crucial to reducing the long-term burden of trauma and poverty (43, 44).

### 2.7 Ethical considerations in trauma care

Ethical trauma-informed care requires respect for individual autonomy, ensuring informed decision-making regarding mental health support (45). Maintaining confidentiality is essential to building trust, though legal limits apply when safety is at risk (46). Cultural sensitivity is also vital: professionals must honor diverse backgrounds and tailor interventions, accordingly, avoiding stereotypes and fostering engagement (47). Equity in access to services must be prioritized to reduce disparities in mental health outcomes (48).

### 2.8 The role of stakeholders

Communities play a foundational role in supporting children's mental health. Parents, caregivers, teachers, and peers can offer emotional support and early intervention through safe, nurturing environments. Educational campaigns and youth programs help destigmatize trauma and promote social inclusion.

Policymakers, health professionals, and non-governmental organizations (NGOs) must prioritize early identification, prevention, and sustained support for trauma-affected youth (49). Policies should integrate mental health services into schools, healthcare, and child protection systems, with adequate resources for implementation (50). Clinicians must provide evidence-based, age-appropriate care such as therapy, cognitive-behavioral interventions, and family counseling while collaborating with schools and community services (51, 52).

NGOs also play a crucial role in delivering direct support, training providers, and advocating for the rights of trauma-affected children and adolescents (53, 54). Together, these stakeholders form the backbone of a trauma-informed ecosystem that can break cycles of suffering and promote lasting recovery and resilience. International conventions safeguarding children in conflict zones are summarized in Table 2, and multi-level recommendations for stakeholders are presented in Table 3.

**Table 2:** Summary of international conventions safeguarding children in conflict zones (55).

S. No.	Legal instrument & year	Key child protection provisions
1	Geneva Convention IV (1949)	Provides special protection for children during international armed conflict, including evacuation to safe zones, care for unaccompanied children, family reunification, and humane treatment.
2	Additional Protocol I (1977)	Strengthens child protection during international conflict by prohibiting recruitment of children under 15, supporting evacuation, and ensuring access to education and cultural continuity.
3	Additional Protocol II (1977)	Extends protections to children in internal conflicts, including the prohibition of recruitment under age 15 and the obligation to provide care, education, and safety.
4	Convention on the Rights of the Child (CRC) (1989)	Calls on states to prevent children under 15 from direct participation in hostilities and emphasizes priority for older youth in any military involvement.
5	Optional Protocol to the CRC on the Involvement of Children in Armed Conflict (OPAC) (2000)	Prohibits the recruitment or use of anyone under 18 in hostilities and requires states to criminalize such practices by armed groups.
6	Rome Statute of the International Criminal Court (ICC) (1998)	Defines the recruitment or use of children under 15 in armed conflict as a war crime and allows for prosecution under international law.

**Table 3:** Multi-level recommendations for supporting children affected by armed conflict.

S. No.	Stakeholder	Key responsibilities	Preparedness & action steps
1	International Organizations (e.g., UN, WHO, UNICEF)	• Establish child-safe spaces (shelters, schools) • Deploy trained personnel for education, healthcare, and protection • Ensure access to mental health services • Enforce international child protection laws	• Train and compensate personnel working in conflict zones • Allocate funds for shelter, food, education, and mental health • Implement mechanisms to prevent trafficking and abuse • Enforce legal accountability for violations • Keep families and sibling groups together
2	National Organizations (Ministries of Health, Child Welfare, Education)	• Deploy trained professionals in crisis settings • Design policies for care of war-affected children • Fund and conduct research on war-related trauma and rehabilitation	• Allocate budgets for emergency child services • Train healthcare professionals and conduct disaster drills • Plan infrastructure and workforce for emergencies • Maintain essential medical supplies • Support research initiatives focused on child recovery
3	Mental Health Professionals	• Provide trauma-informed care • Support other frontline workers • Advocate and Information policy • Lead community recovery efforts	• Build ethical and moral resilience • Train in triage, assessment, psychological first aid, therapy • Stay updated on trauma interventions and pharmacotherapy
4	Healthcare Workers	• Deliver medical and psychological care to children • Document and report ethically • Ensure continuity of care	• Undergo disaster response drills • Receive resilience and trauma-response training • Get access to mental health support and supervision
5	Teachers & Community Resources	• Identify and support at-risk children • Offer emotional reassurance and connection • Educate children on safety and available resources	• Raise awareness of war's impact on children • Create local resource centers with food, shelter, and basic services • Promote inclusion and social support in schools and communities

## 3 Discussion

### 3.1 Principal findings

This review synthesizes evidence indicating that armed conflict and forced displacement exert profound, multidimensional, and durable effects on the psychological well-being of children and adolescents. Across the conflict trajectory, exposure is consistently associated with elevated rates of PTSD, depression, anxiety, behavioral dysregulation, sleep disturbance, and impaired social and educational functioning (10, 17, 22). Meta-analytic evidence indicates that PTSD and depression are substantially more common among war-affected populations than among non-exposed populations. However, many of these meta-analyses include predominantly adult or mixed-age samples rather than exclusively children and adolescents. Accordingly, these findings should be interpreted as reflecting the broader psychiatric burden associated with armed conflict rather than direct prevalence estimates for conflict-affected youth. Studies focusing specifically on children and adolescents consistently report elevated rates of PTSD, depression, anxiety, and behavioral difficulties, although prevalence estimates vary considerably according to conflict setting, displacement status, timing of assessment, and measurement instruments (21, 22). Critically, the harms of conflict are not confined to the acute phase: longitudinal evidence demonstrates that mental health sequelae frequently persist into adolescence and adulthood and may be transmitted to subsequent generations through psychological, familial, and social pathways, with biological mechanisms proposed but not established (23, 24, 29, 62).

Three cross-cutting patterns emerge from the synthesized literature. First, the timing and chronicity of exposure matter as much as its severity prolonged or repeated exposure during sensitive developmental windows produces compounding effects that brief, single-incident trauma frameworks do not adequately capture (18, 31). Second, outcomes are shaped less by exposure alone than by the post-exposure environment, particularly the availability of stable caregiving, educational continuity, and culturally embedded social support (10, 11, 40). Third, mental health outcomes cannot be disentangled from concurrent deprivations in nutrition, healthcare, safety, and economic opportunity; conflict produces a syndemic burden in which psychological and structural harms reinforce one another (9, 27).

### 3.2 Mechanisms of harm across levels of analysis

Understanding why conflict exposure produces such pervasive psychological consequences requires integrating evidence across biological, psychological, and social levels. At the neurobiological level, chronic and severe early-life stress disrupts the development of corticolimbic circuits implicated in threat appraisal, emotion regulation, and executive function, with documented alterations in amygdala–prefrontal connectivity, hypothalamic–pituitary–adrenal axis reactivity, and stress-related gene expression (23, 24, 66). Epigenetic studies have reported differences in DNA methylation at glucocorticoid-related loci in trauma-exposed individuals and in their offspring, and this has been proposed as one contributor to intergenerational risk (24). That literature remains preliminary and contested. Human studies are small, largely cross-sectional, restricted to a narrow set of candidate loci, and unable to separate germline transmission from prenatal exposure, postnatal caregiving, and shared environment, while the most controlled evidence comes from animal models and does not translate directly to human populations (23, 61). The intergenerational patterning of risk observed in clinical and epidemiological data is at present better accounted for by psychological, familial, and social transmission than by inherited epigenetic change (62).

At the psychological level, conflict exposure disrupts core developmental tasks. For young children, traumatic separation and caregiver unavailability undermine the formation of secure attachment, a foundational substrate for later emotion regulation and interpersonal functioning (19). For school-aged children and adolescents, the loss of predictable routines, peer networks, and educational structure interferes with identity formation, moral development, and the acquisition of cognitive and socioemotional skills (22, 35, 36). The constructs commonly used to describe these effects PTSD, depression, anxiety capture only part of the clinical picture; complex post-traumatic presentations, traumatic grief, somatic distress, and culturally specific idioms of distress are frequently reported but inconsistently measured (13, 17).

At the social and structural level, Bronfenbrenner's ecological framework helps explain how conflict reaches the child through cascading disruptions to family, school, community, and societal systems (60), a model that has been explicitly applied to war-affected youth (5, 18). Caregiver mental health, family cohesion, peer relationships, school climate, and community resources each independently predict child outcomes, and the simultaneous degradation of these systems during conflict amplifies risk in non-additive ways (10, 18). This framework underscores why narrowly clinical interventions, however evidence-based, are insufficient in the absence of broader system-level repair.

### 3.3 What works: the evidence base on interventions

The interventions evaluated in this population differ substantially in the strength of the evidence supporting them, and presenting them as a single undifferentiated portfolio obscures the choices that clinicians and program planners actually face. The strongest evidence supports trauma-focused cognitive behavioral therapy (TF-CBT) and narrative exposure therapy adapted for children (KIDNET). Both have been tested in randomized trials with conflict-affected and refugee youth, and both produce moderate to large reductions in PTSD symptoms in meta-analyses spanning diverse settings (12, 56). Direct head-to-head comparisons remain scarce and do not establish the superiority of either approach (56), so the choice between them rests more on delivery constraints than on relative efficacy. TF-CBT typically requires eight to sixteen sessions and structured caregiver involvement, whereas KIDNET can be delivered in roughly four to ten sessions and does not depend on an available caregiver, an advantage where family structures have been destroyed or separated (11, 56).

An intermediate tier comprises school-based and group-delivered programs such as Teaching Recovery Techniques, Writing for Recovery, and classroom-based mind and body interventions. These have been evaluated in controlled trials, but the results are more variable and the pooled picture is more nuanced than summary statements suggest (10, 11). An individual participant data meta-analysis of focused psychosocial interventions in low-resource humanitarian settings found overall benefit for PTSD symptoms and functional impairment as well as for hope, coping, and social support, but the improvement in PTSD symptoms was concentrated among adolescents aged 15 to 18 years, among non-displaced children, and among children in smaller households, and was attenuated in younger and displaced children (63). That heterogeneity matters clinically, because the subgroups showing least benefit are frequently the ones humanitarian programs are designed to reach. Analyses of common practice elements further suggest that much of the benefit of these programs derives from a shared core of psychoeducation, affect regulation, cognitive restructuring, narrative or exposure work, and social support building rather than from any single branded protocol, which has direct implications for what must be preserved when a program is shortened, translated, or task-shifted (64).

A third tier comprises creative arts and expressive modalities, caregiver-focused parenting interventions targeting parental mental health and dyadic regulation, and community-level psychosocial activities such as child-friendly spaces. These rest on a thinner base of small trials, uncontrolled pre-post evaluations, and qualitative work (53, 64). They are widely implemented within the Inter-Agency Standing Committee (IASC) tiered model and are valued for acceptability, reach, and low delivery cost, but the current evidence supports them as plausible components of a layered response rather than as established treatments for diagnosable disorders (53, 54, 57).

Comparative efficacy is only one axis of the decision, and in this field it runs counter to the others. The modalities with the strongest evidence are the most specialist-dependent, requiring trained therapists, clinical supervision, session continuity, and a degree of physical safety that active conflict rarely permits, whereas the modalities with weaker evidence are precisely those that teachers, community health workers, and trained non-specialists can deliver at population scale (53, 54, 63). Cultural adaptation introduces a further tension. Surface adaptations such as translation, locally contextualized examples, and culturally acceptable delivery settings are routinely undertaken and do not appear to compromise effects, whereas deeper adaptations to explanatory models of distress, help-seeking norms, family decision-making, and the acceptability of narrating traumatic experience aloud are less often documented and almost never evaluated as adaptations in their own right (10, 47, 48). Implementation constraints in active conflict compound both problems: attrition from renewed displacement, insecurity for participants and providers alike, absent referral pathways for children who deteriorate during treatment, humanitarian funding cycles that end programs before gains consolidate, and the ethical difficulty of initiating exposure-based work while the traumatic environment is ongoing rather than past (54, 57). Taken together, these considerations favor stepped and layered delivery, in which non-specialist and group-based approaches provide broad coverage while scarce specialist capacity is reserved for children with severe or persistent impairment, an approach consistent with the mhGAP Humanitarian Intervention Guide but not yet adequately tested at scale (58). Table 4 summarizes these comparisons across the main intervention categories.

**Table 4:** Comparative summary of interventions for children and adolescents affected by armed conflict and forced displacement.

Intervention category	Strength of evidence	Typical delivery requirements	Scalability in active conflict	Principal constraints
Trauma-focused cognitive behavioral therapy (TF-CBT)	Strong. Multiple randomized trials and meta-analyses in conflict-affected and refugee youth; moderate to large effects on PTSD symptoms (12, 56)	Eight to sixteen sessions; trained therapist; ongoing supervision; structured caregiver involvement	Low	Specialist workforce, session continuity, caregiver availability, physical safety over months
Narrative exposure therapy for children (KIDNET)	Strong. Randomized trials and meta-analyses; comparable to TF-CBT where directly compared (12, 56)	Roughly four to ten sessions; trained provider; no caregiver requirement	Low to moderate	Supervision needs; ethical and clinical difficulty of narrative exposure while threat is ongoing
School-based group programs (Teaching Recovery Techniques, Writing for Recovery)	Moderate. Controlled trials with heterogeneous effects; benefit concentrated in adolescents aged 15 to 18 years, non-displaced children, and smaller households (11, 63, 64)	Group format; teacher or non-specialist facilitator after brief training and supervision	High where schools are functioning	Requires functioning schools; weaker effects in younger and displaced children; attrition with renewed displacement
Mind and body and creative arts modalities	Limited. Small trials and uncontrolled pre-post evaluations; outcome measures heterogeneous (53, 63)	Group format; lay or para-professional facilitator; minimal materials	High	Unclear durability; weak comparative data; effects rarely assessed against active controls
Caregiver and parenting interventions	Emerging. Promising for caregiver mental health; child outcomes less consistent (10, 53)	Group or home-based sessions; trained non-specialist	Moderate	Caregiver availability and engagement; competing survival demands; caregiver mortality and separation
Community-level psychosocial activities (child-friendly spaces)	Limited. Largely programmatic and descriptive evaluation rather than controlled trials (53, 57)	Community volunteers; brief training; safe physical space	High	Not a treatment for diagnosable disorder; weak outcome data; quality varies widely across sites
Pharmacotherapy	Very limited in this population; largely extrapolated from non-conflict pediatric samples (58)	Prescriber, reliable supply chain, monitoring capacity	Very low	Supply continuity, monitoring and follow-up capacity, limited pediatric evidence in humanitarian settings

TF-CBT, trauma-focused cognitive behavioral therapy; KIDNET, narrative exposure therapy adapted for children; PTSD, post-traumatic stress disorder. Bracketed numerals refer to the reference list. Strength of evidence reflects study design, replication, and consistency of effects in conflict-affected and refugee samples specifically, rather than in general pediatric populations.

Despite this progress, the intervention evidence base has substantial gaps. Most trials are short in duration, evaluate symptom reduction rather than functional or developmental outcomes, and are conducted in a narrow set of contexts predominantly resettled refugee samples in high-income countries or selected post-conflict settings limiting generalizability to children in active conflict zones, in protracted displacement, or in under-studied conflicts (10, 11, 17). Few studies directly compare modalities, examine optimal sequencing or stepped-care approaches, or assess sustainability after withdrawal of external funding. Evidence for pharmacological treatment in this population is thinner still, is largely extrapolated from non-conflict pediatric samples, and rarely informs frontline humanitarian practice (58).

### 3.4 Equity, access, and the global mental health treatment gap

Even where effective interventions exist, the treatment gap for conflict-affected children remains profound. In many LMICs hosting most displaced children, child and adolescent mental health specialists number in the single digits per million population, and dedicated child-focused services within humanitarian responses are routinely under-prioritized and under-funded relative to physical health and shelter (49, 53). Task-shifting and task-sharing models that train non-specialist providers community health workers, teachers, and refugee peer workers to deliver structured psychosocial interventions are a pragmatic response and have shown feasibility and acceptability, but their effectiveness, safety, and supervisory requirements at scale remain incompletely characterized (53, 54). The WHO–UNHCR mhGAP Humanitarian Intervention Guide provides a widely adopted framework for non-specialist clinical management of priority mental health conditions in humanitarian emergencies and has been adapted across multiple conflict-affected settings (58).

Equity concerns also extend beyond geography. Adolescent girls, children with disabilities, unaccompanied and separated minors, child soldiers and survivors of sexual violence, and children from stigmatized ethnic or religious groups face compounded barriers to identification and care yet are inconsistently represented in research samples and intervention trials (20, 25). Digital and remote mental health delivery has expanded rapidly and may help address geographic and workforce constraints, but raises distinct concerns about privacy, connectivity, language adaptation, and the appropriateness of self-guided tools for severely traumatized youth.

### 3.5 Methodological limitations of the existing literature

Several methodological constraints temper the strength of inferences that can be drawn from the existing evidence. First, most studies are cross-sectional, limiting causal interpretation and obscuring developmental trajectories; the relatively few longitudinal cohorts that exist are heterogeneous in follow-up duration, attrition handling, and outcome ascertainment. Second, assessment of psychopathology relies overwhelmingly on Western-developed, symptom-based instruments that may have limited cross-cultural validity and may under- or over-capture distress in populations whose phenomenology and idioms of suffering differ from those in which the instruments were developed. Third, control comparisons are inherently difficult in conflict settings, and confounding by pre-existing adversity, concurrent deprivation, and selection into help-seeking is rarely fully addressed. Fourth, the body of evidence is geographically concentrated, with disproportionate representation of a small number of conflicts and under-representation of protracted, lower-visibility crises. Finally, the perspectives of children, adolescents, and their caregivers are infrequently centered in study design or interpretation, even when they are the subjects of the research.

### 3.6 Strengths and limitations of this review

This review offers a structured synthesis of psychological outcomes, mechanisms, protective factors, and intervention considerations across the temporal arc of conflict exposure, integrating clinical, developmental, and structural perspectives that are often discussed in isolation. Limitations include the non-systematic nature of the search, which prioritized representative and thematically rich literature over exhaustive coverage; the restriction to English-language sources, which may bias the evidence base toward certain regions and academic traditions; and the absence of a formal risk-of-bias appraisal or quantitative pooling of effect estimates. Heterogeneity in study designs, populations, exposure histories, instruments, and definitional conventions also constrains direct comparability across the included studies. The synthesis presented here should therefore be understood as a conceptual map intended to orient clinicians, researchers, and policymakers, rather than as a definitive quantitative summary.

### 3.7 Implications for policy and practice

Several policy and practice implications follow from the evidence reviewed. First, mental health and psychosocial support must be treated as a core component of humanitarian response rather than an optional add-on, with dedicated funding lines, indicators, and accountability mechanisms aligned with the IASC guidelines and the Sphere standards (57, 59). Second, child-focused services should be integrated across the sectors that already reach primary healthcare, schools, child protection, and community programs—rather than concentrated in stand-alone specialty clinics that few children can access (51, 52, 58). Third, investments in workforce development are essential and should include training and ongoing supervision for non-specialist providers, protection of frontline clinicians’ own well-being, and pathways for refugee and host-community practitioners to enter and remain in the workforce (53, 54). Fourth, durable solutions for displaced children, safe family reunification, regularized legal status, school enrollment, and economic inclusion of caregivers are themselves mental health interventions, and policies that prolong precarity should be recognized as health-harming. Finally, the rights-based framework articulated in the international conventions summarized in Table 2 should anchor advocacy, accountability, and the design of services for war-affected youth.

### 3.8 Future research directions

Priority research directions include: (i) prospective, longitudinal cohort studies that track developmental trajectories from acute exposure through adulthood and across generations, including biological markers of stress and resilience; (ii) cultural adaptation and validation of assessment tools that can capture both universal and context-specific expressions of distress; (iii) pragmatic trials evaluating stepped-care, task-shifted, and digitally augmented interventions at scale, with attention to functional and developmental outcomes rather than symptom reduction alone; (iv) implementation research examining how evidence-based interventions are delivered, sustained, and adapted within humanitarian systems; (v) studies that explicitly include under-represented populations, including child soldiers, survivors of conflict-related sexual violence, children with disabilities, and youth from protracted and low-visibility conflicts; and (vi) participatory research that meaningfully involves children, adolescents, caregivers, and affected communities in defining priorities, designing interventions, and interpreting findings. Closing these gaps will require sustained funding, equitable global research partnerships, and durable institutional commitments that match the scale of the population in need.

### 3.9 Synthesis

Taken together, the evidence reviewed argues for a shift away from narrow, deficit-focused, single-modality approaches toward a sustained, interdisciplinary, and rights-based response to the mental health needs of children affected by armed conflict and forced displacement. Such a response must be embedded within schools, families, healthcare systems, and child protection frameworks; must center the agency, dignity, and cultural worlds of affected children; and must hold political and humanitarian actors accountable for the conditions that produce harm in the first place. Mental health professionals, working alongside educators, community leaders, policymakers, and the children and families they serve, are well positioned to lead this work if scale, equity, and durability are treated not as aspirational ideals but as core measures of success.

## 4 Conclusion

Armed conflict imposes profound and lasting psychological and developmental harms on children and adolescents, extending far beyond immediate exposure to violence and displacement. These effects include chronic mental health disorders, disrupted education, and intergenerational consequences that are often overlooked in global policy responses. Despite growing awareness, existing mental health and psychosocial interventions remain fragmented, underfunded, and inconsistently implemented. This review underscores the urgent need for trauma-informed, culturally responsive, and scalable interventions embedded within schools, families, and communities. Coordinated, rights-based action is essential to ensure that resilience is supported through sustained investment, allowing war-affected children not only to survive, but to heal and thrive.
